# “Clinical pharmacognosy”- A new interesting era of pharmacy in the third millennium

**DOI:** 10.1186/2008-2231-20-18

**Published:** 2012-08-30

**Authors:** Mahnaz Kazemi, Azadeh Eshraghi, Afsaneh Yegdaneh, Alireza Ghannadi

**Affiliations:** 1 Department of Pharmacognosy, School of Pharmacy and Pharmaceutical Sciences and Isfahan Pharmaceutical Sciences Research Center, Isfahan University of Medical Sciences, Isfahan, Iran; 2 Department of Clinical Pharmacy, Faculty of Pharmacy, Shahid Beheshti University of Medical Sciences, Tehran, Iran

## 

Pharmacy is known as a branch related to healthcare services [1]. According to the features of pharmacognosy and clinical pharmacy two distinctive and important subjects of pharmacy in this editorial it has been introduced as a new integrated and multidisciplinary feature between these two subjects named; “clinical pharmacognosy”.

Pharmacognosy, which literally means studying medications of natural sources, has been a part of medical arts and sciences since mankind first began to treat illnesses [2]. To get a proper perspective about this science, which deals with plant, animal, mineral and other natural medications, it is extremely helpful to investigate the historical aspects of this science and to recognize the pioneers of this field. By an experience obtained from trial and error, early man had to acquire biologic knowledge useful in determining the effects of available foods and beverages derived from plants. Although the pharmacognosy term was used for the first time in 1811 by the Austrian physician, Schimdt, the founder of the first independent pharmacognosy institute in the world was Crovatian Domac in 1896 [2-4]. In the field of pharmacognosy the Persian outstanding scientists like Rhazes, Avicenna and Jorjani created major progresses and improvements that started modern pharmacognosy in the world. Fortunately traditional medicine and its remedies contribute to a major role in the current health care systems of several countries [4,5].

The study of medications of plant origin includes the subjects of botany, chemistry and pharmacology. Botany includes toxonomy, genetics, and cultivation of plants. Chemical characterization includes the isolation, elucidation and quantification of constituents and natural products pharmacology. The science of pharmacognosy has been progressed to phytochemistry, plant biotechnology, medical ethnobotany, ethnopharmacology, phytotherapy, marine and zoo pharmacognosy. Although pharmacognosy has been improved and expanded in several aspects, it has no hard link with clinical trials yet [2,5].

Clinical pharmacy requires optimum use of medications, therapeutic knowledge, counseling, clinical experience, therapeutic drug monitoring and disease good diagnosis. A clinical pharmacist collaborates with physicians in multidisciplinary meetings and rounds and would advise regarding the cost-effectiveness, safety and appropriateness of medications. Clinical pharmacists should monitor patient outcomes according to the patient’s situation and the probable risks associated with therapy. Recognition of therapeutic systems and patient records are principles of this field. Clinical pharmacists should prepare a nice atmosphere and consultation area and have a good communication skill as well as should know the patient’s quality of life that can be obtained via interview of patients [1,6,7]. Also the clinical pharmacist should be aware of disease etiology, drug allergies, drug interactions and patient monitoring to choose the rational drug and regimen. Therefore the clinical pharmacist should know to detect and document adverse reactions to recommend selecting better choice [1,7].

While clinical pharmacy significantly progressed, a gap between this science and herbal and traditional medicines fields is evident. As the use of herbals increased in the recent years, new kinds of demands are raised. There are several potential problems associated with herbal remedies like lacking of systematic reviews and evidence-based data about their efficacies [1,2,5,7]. There are a few systematic reviews about traditional Iranian medicine [8-12].

One of the reasons behind increase in use of herbal medicines is the belief that they have lower toxicities but the fact is that herbal medicines may cause unwanted effects, allergic or toxic reactions [2,5,13]. Herbs can even cause drug-drug, drug-food and herb-drug interactions if not properly used specially in use of OTC drugs [5,13,14].

Clinical pharmacognosy should deal with above problems and find the best solutions for them. Although this term has been used in a workshop in the United States entitled, “Clinical Pharmacognosy: Contribution of pharmacognosy to clinical trials of botanicals and dietary supplements”, which took place at the American Society of Pharmacognosy meeting in Portland, Maine, on July 2007 and in a Japanese journal on March 2011, there has not been any comprehensive definition yet [15,16]. The renaissance of herbal medicine in the world creates a demand for studies in the field of pharmacognosy, traditional medicine and some related fields. From a practical perspective this includes quality control (identity, purity, consistency), efficacy (therapeutic indications, clinical studies, pharmacological investigations), documentation and safety (adverse reactions, drug interactions, contraindications, precautions and toxicities). There are huge capacities of research topics in the herbal and traditional medicine fields which this new discipline can design and perform them clinically. This new borderline discipline may extend the scope of clinical aspects of pharmacognosy and play a progressive role in the safe, rational and efficient use of traditional and herbal medicine. There are several unproven therapeutic benefits and undisclosed toxicities and difficulties in standardizing natural treatments [2,13-16]. Clinical pharmacognosy is a bridge between clinical research and botanicals knowledge providing clinical and pharmaceutical researchers, physicians and other healthcare professionals with the key information they need to assist the progress of herbal and traditional medicines. Clinical pharmacognosist should ask the patients about the herbal drugs or other supplements they have had taken before and about the history of possible allergic reactions. He should also evaluate the patient recovery process after using each kind of synthetic, herbal or traditional drugs and should focus on resolving a wide range of challenging problems [8,11,12,15,16].

It seems that in the third millennium, we need education of clinical pharmacognosists for providing more details regarding different aspects of clinical applications of natural health products. In order to use rational herbal and traditional medicines and adding standard clinical values to them, establishing and spreading of clinical pharmacognosy feature may help to improve health to all. Clinical pharmacognosist can provide full and correct information about all pharmaceutical and medical aspects of plants, natural health products and dietary supplements. This field may plays leading and interesting roles in identifying, analyzing, standardizing, controlling, documentation and determining of these natural health products evidence based medicines. It increases more the findings of effective naturals especially by doing systematic review of randomized controlled trials evaluated herbal therapies for different diseases [2,5,14-16]. For instance, a recent systematic review in traditional Iranian medicine (TIM) opened a new era in the field of inflammatory bowel disease and has introduced new research lines [17]. Interestingly, in TIM, name of diseases or even herbal compounds have some differences with the present names and terms; so researchers must be so careful to study TIM and translate it into current language. New attention to these effective herbs will lead us to discovery and obtaining novel natural drugs.

## Authors’ contributions

All authors contributed to the review of the literature and design and writing of the manuscript. They have read and approved the manuscript content.
